# Beyond hearing loss: exploring neurological and neurodevelopmental sequelae in asymptomatic congenital cytomegalovirus infection

**DOI:** 10.1038/s41390-025-04232-5

**Published:** 2025-07-08

**Authors:** Meghan R. Swanson, Lauren D. Haisley, William B. Dobyns, Mark R. Schleiss

**Affiliations:** 1 Masonic Institute for the Developing Brain, Center for Neurobehavioral Development, and Institute for Child Development, Division of Clinical Behavioral Neuroscience, University of Minnesota Medical School Minneapolis, Minneapolis, MN USA; 2 Division of Pediatric Genetics & Metabolism, Department of Pediatrics, University of Minnesota Medical School Minneapolis, Minneapolis, MN USA; 3 Division of Pediatric Infectious Diseases, University of Minnesota Medical School Minneapolis, Minneapolis, MN USA

## Abstract

**Abstract:**

Congenital cytomegalovirus (cCMV) infection is common, and usually clinically inapparent. The prevalence of infection is approximately 1:200 births, but only 10–15% of infants have clinically apparent CMV disease (CACMV) as newborns. The most common long-term disability is sensorineural hearing loss (SNHL), which occurs in 10–15% of all cases. Infants with CACMV are also at increased risk for intellectual disability, cerebral palsy, learning disabilities, ocular and cortical blindness, seizure disorders, developmental delay, and autism spectrum disorders. Although infants with clinically inapparent cCMV (CICMV) are at risk for SNHL, the spectrum of other adverse neurodevelopmental outcomes is less clear, since few studies have tracked neurodevelopment in this setting. With the advent of universal cCMV screening, most cCMV infections will now be identified in infants with CICMV. These infants require serial audiologic monitoring, but many questions are unanswered, including what kinds of diagnostic evaluations are required; what kinds of central nervous system (CNS) imaging studies are recommended; what the utility and value of developmental assessments is; and whether there are biomarkers that can inform the long-term prognosis and direct anticipatory guidance in monitoring for neurologic and neurodevelopmental adverse outcomes.

**Impact:**

- Universal newborn screening for congenital CMV (cCMV) infection has been implemented in many US states and Canadian provinces.
- Most infants identified by universal screening have CICMV infections. All require audiologic monitoring, but there is minimal experience to direct other evaluations, including laboratory tests, brain imaging and neurodevelopmental assessments.
- Adverse neurodevelopmental outcomes in CICMV may be more extensive than previously appreciated. Research is needed to define the full range of potential neurocognitive disability. New knowledge generated by studying CICMV infections may aid in reclassification of the scope of disease in an emerging era of universal cCMV screening.

## Introduction

### Congenital Cytomegalovirus Infection is an Urgent Public Health Problem

The most common infectious cause of neurodevelopmental disability in the United States (US) and Europe, and probably globally, is congenital cytomegalovirus (cCMV) infection. The injury induced by this virus is largely dependent upon the timing of fetal acquisition of the infection, with the first trimester of pregnancy representing the highest risk window.^1–7^ Overall cCMV prevalence in various studies ranges from 0.2% to 2.5%.^8,9^ A meta-analysis published in 2007 estimated an overall prevalence of 0.64%, but noted that large geographic and demographic variability has been reported.^10^ A more recent study estimated a pooled overall global prevalence of cCMV of 0.67%, ranging from 0.48% in high-income countries to 1.42% in low- and middle-income countries.^11^ Seropositivity rates are higher in these populations,^12^ and maternal-fetal transmission is known to be directly proportional to maternal seroprevalence.^13^ Notably, cCMV infection is a disease of health disparities, disproportionately impacting black and multiracial infants.^14–18^ Currently no licensed vaccine to prevent cCMV exists, and in spite of its importance to newborn health, most women of childbearing age are not aware of the risks of infection.^19,20^ Currently no systematic pre-natal screening for CMV is done, and little information is provided to patients by women’s health practitioners about strategies to avoid infection.^21^ Given the magnitude of disability caused in childhood by cCMV, the infection can be considered a major and unsolved public health problem.

## Congenital CMV: disease definitions and sequelae

Infants with cCMV are at risk for neurologic and developmental disabilities. The overall likelihood of CMV-specific symptoms at birth (also referred to as “symptomatic cCMV”) was estimated to be 12.7% in one meta-analysis.^8^ The overall percentage of these children who had long-term sequelae was 40–58%. Long-term sequelae are most common in infants that are symptomatic (obvious CMV disease at birth)^22^ but can be observed in asymptomatic congenital infections as well. Among the 87.3% of cCMV infants who did not have obvious evidence of disease at birth, 13.5% developed long-term sequalae, with almost all of this risk attributed to sensorineural hearing loss (SNHL).^8^ We define cCMV cases with no obvious evidence of disease at birth as **clinically inapparent cCMV (CICMV) infections**.

The risk of SNHL in CICMV infection is substantial and represents a major driving force behind implementation of newborn CMV screening programs. Since SNHL occurs in children with cCMV in the absence of other clinical signs of disease, such infections are often overlooked. Reliance on universal newborn hearing screening (NHS) to capture these children is inadequate, since over 40% of pediatric SNHL due to cCMV infection is not present at birth but rather is delayed in onset. Hence, these children will be missed in the newborn nursery by NHS alone, absent cCMV screening.^23–26^

**Fig. 1:**
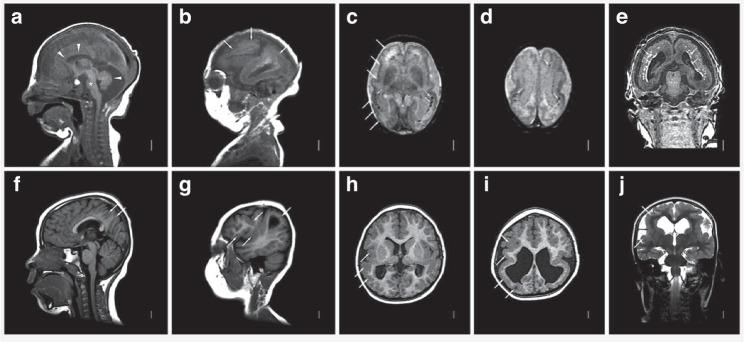
MRI scan findings in CACMV. This image demonstrates brain MRI abnormalities observed in cCMV. Images from a child with CACMV (panels **a**–**e**) demonstrate diffuse thin corpus callosum (upper arrowheads in **a**), small pons (asterisk in **a**), diffuse cerebellar hypoplasia (lower arrowhead in **a**), diffuse reduced number and complexity of gyri and irregular cortex consistent with PMG seen in all regions (arrows in **b**, long white arrows shown only on the right in **c**), and extensive periventricular and subcortical white matter injury and calcifications (short white arrow in **c**–**e**), and mild-moderate ventriculomegaly. Images from another patient with CACMV show normal corpus callosum and cerebellum with PMG over the parietal convexity and right perisylvian region (arrows in **f**, **g**), bilateral perisylvian predominant PMG (long white arrow shown only on the right in (**h**–**j**), and moderate posterior ventriculomegaly. The images include T1-weighted sagittal (**a**, **b**, **f**, **g**), T2-weighted non-turbo axial (**c**, **d**), 3D-MPRAGE coronal (**e**) and axial (**h**, **i**) and T2-weighted coronal (**j**) sequences.

With the exception of SNHL, the risks of other long-term neurodevelopmental disabilities in CICMV infection are not well-defined. In contrast, infants with **clinically apparent evidence of cCMV disease (CACMV)** in the newborn period have high rates of neurologic and neurodevelopmental disability. Signs or symptoms at birth that trigger concern for CACMV include hepatosplenomegaly, jaundice, thrombocytopenia, microcephaly, neurological findings including seizures, and hearing and vision deficits. Maternal history is also an important variable, with histories of symptomatic maternal infection, an abnormal fetal ultrasound, and/or an abnormal prenatal MRI or both serving as important clues.^27^ A wide range of fetal/neonatal neurologic injuries are attributable to cCMV, including microcephaly, cortical malformations especially polymicrogyria (PMG), hydrocephalus, cerebral calcifications, cerebellar hypoplasia, hypomyelination, and retinitis (Fig. 1). Among children with CACMV at birth, studies report risks of 40–71% that these children will go on to develop permanent infection-related sequelae, including SNHL, developmental delay and neurologic handicaps.^8,28,29^ Overall, the risk of long-term impairment in all children with cCMV (both CICMV and CACMV) is approximately 25%.^29^ As noted above, symptomatic disease and long-term sequelae are more common when fetal infection is acquired in the first trimester of pregnancy, particularly in the context of a maternal primary CMV infection,^5^ although congenital transmission in the context of nonprimary maternal infections also contributes to the burden of childhood cCMV disease.^30^

Variability in both the reported percentages and sampling techniques used in quantifying the risk of long-term sequelae in infants with cCMV is in no small part a reflection of the variable characteristics that define “asymptomatic” and “symptomatic” cCMV infection.^31^ The definitions of what constitutes “asymptomatic” and “symptomatic” status at birth have evolved, and currently are based not only on clinical findings, but also on laboratory tests, neuroimaging, audiologic, and ophthalmologic evaluations.^32^ The nomenclature in the cCMV field is challenging. For example, a child with CICMV may nonetheless be classified as “symptomatic” depending upon the results of diagnostic evaluation. The over-arching challenge in the field is that infants with CICMV have not routinely undergone comprehensive diagnostic evaluation, precisely because clinicians are unaware of these infections. After all, why would an asymptomatic, healthy-appearing newborn be tested for cCMV? That paradigm is changing because of the advent of universal cCMV screening.

A commonly used definition of cCMV disease category comes from an international consensus panel.^33^ In this algorithm, SNHL is not included as a disease-defining manifestation absent other abnormalities, such that neonates with isolated SNHL are classified as “asymptomatic” infection. This nomenclature, although well-intended, is often confusing to clinicians. These definitions are in flux, and more recent European expert consensus statements have included cCMV-associated SNHL as a defining characteristic of “symptomatic” congenital infection.^31,34,35^ Table 1 outlines the widely used disease classification categories as published by Rawlinson et al. Disease classification is important for clinical decision-making about the use of antiviral therapy. Ideally, in the future an international consensus statement can be pursued as an area of high priority. Although most clinical decision-making emphasizes the terms “symptomatic” and “asymptomatic”, we recommend an approach from more recent studies that uses the terminology of CACMV and CICMV infections.^36,37^ A key issue that will be critical to resolve is the question of whether CICMV infants are at risk for neurodevelopmental sequelae beyond SNHL. Although laboratory and neuroimaging evaluations may define CICMV infections as “symptomatic” cCMV disease, even in the absence of any identifiable concerns in the antenatal history or newborn examination, our knowledge about long-term outcomes for infants in this population is incomplete. This is a particularly important consideration in the emerging era of universal newborn screening programs.

**Table 1:** cCMV disease classification.

**Definitions of cCMV Disease Categories**^**a**^	
**Symptomatic cCMV Infection with CMV Disease/Clinically Apparent cCMV (CACMV)**	
Mildly Symptomatic cCMV Disease	• Defined as cCMV infection with one or two isolated disease manifestations. • Clinical or laboratory findings are mild and transient (eg, mild hepatomegaly; a single measurement of low platelet count or elevated transaminases). • No evidence of neuroimaging abnormalities or SNHL.
Moderately-to-Severely Symptomatic cCMV Disease	• Multiple disease manifestations attributable to cCMV: thrombocytopenia, petechiae, hepatomegaly, splenomegaly, intrauterine growth restriction, or hepatitis (raised transaminases or bilirubin). • CNS involvement including microcephaly, radiographic abnormalities (ventriculomegaly, intracerebral calcifications, periventricular echogenicity, cortical or cerebellar malformations), abnormal cerebrospinal fluid findings including detection of CMV DNA in cerebrospinal fluid, chorioretinitis, SNHL.
**Asymptomatic cCMV Infection/Clinically Inapparent cCMV (CICMV)**	
Asymptomatic cCMV Disease with Isolated SNHL	• No apparent abnormalities to suggest cCMV disease, but sensorineural hearing loss (≥21 decibels) detected by audiological evaluation.
Asymptomatic cCMV	• No apparent abnormalities to suggest cCMV disease, and documentation of normal hearing by audiologic evaluation.

^**a**^After Rawlinson et al., Congenital cytomegalovirus infection in pregnancy and the neonate: consensus recommendations for prevention, diagnosis, and therapy.^33^

## Universal newborn cCMV screening: challenges and opportunities

Recently, universal cCMV screening, based on enhancements in test performance^38^ for PCR detection of CMV DNA in studies conducted using newborn dried blood spot (DBS) cards, has either been implemented or is planned in several US States, including Minnesota, New York, and Connecticut.^39,40^ In addition, universal screening has been implemented in Ontario, Canada, and is planned for the provinces of Saskatchewan and Alberta.^41^ Robust debates about the wisdom of universal CMV screening are ongoing,^32,42^ including the concern that such screening could generate a “vulnerable child syndrome” for infants believed to have a favorable prognosis. Overall, we believe that implementation of universal screening is an exciting and positive development in public health. An unresolved issue is the question of how to best manage newborns identified by universal cCMV screening who have a CICMV infection. A consensus has emerged that these infants require serial audiological monitoring, given the risk of delayed-onset SNHL that may not be manifest at the time of the NHS. However, we have little knowledge to inform and direct clinical management of these infants beyond the need for ongoing evaluation by an audiologist precisely because universal screening programs have only recently been begun. The current recommendations from the Minnesota Department of Health, implemented when universal cCMV screening was commenced in 2023, are summarized in Table 2. But these recommendations are informed in large measure by practice guidelines that have been drawn from clinical experience with CACMV infections from the pre-universal screening era.^33–35^ The key issues that require resolution and additional research are considered below.

**Table 2:** cCMV diagnostic evaluation for infants with positive newborn screen.

**Minnesota Department of Health (MDH) Recommendations for Clinical Evaluations of Infants with CMV Detected by Newborn Screening**^**a,b**^
**Clinical Assessment**
History and physical examination with particular attention to the following • Maternal history of primary CMV infection, prenatal ultrasonography abnormalities. • Assess if infant was born small for gestational age or has microcephaly. • Palpation for hepatomegaly, splenomegaly, or both. • Skin exam for jaundice, petechiae, purpura, blueberry muffin rash. • Neurologic assessment of muscle tone, abnormal reflexes, or other neurologic findings.
**Laboratory and Imaging Assessment**
• Confirmation of cCMV by urine CMV PCR prior to 21 days of age. • Collection of complete blood count (CBC) with differential leukocyte count and platelet count. • Hepatic function tests (transaminase, serum bilirubin). • Cranial ultrasound. A brain MRI may be recommended in some settings (see text for details).
**Additional Assessments**
• Audiology evaluation. Initial audiologic examination including auditory brainstem response (ABR) studies irrespective of results of newborn hearing screening examination, followed by regularly scheduled audiology to monitor for delayed onset/progressive hearing loss. • Ophthalmology examination for baseline visual assessment for eye findings (e.g., chorioretinitis) associated with cCMV.

^a^Modified from MDH cCMV newborn screening program clinical practice guideline published at: https://www.health.state.mn.us/people/newbornscreening/program/cmv/followup.html.

^b^Newborn screening is performed on infant dried blood spot PCR testing for CMV DNA; all positive cases should have a confirmatory urine sample for CMV PCR obtained to confirm diagnosis of cCMV prior to engaging in additional diagnostic evaluation.^90^

### Pathogenesis

The pathogenesis of CNS injury in the context of both CICMV and CACMV is incompletely understood but has been described in several reviews.^7,43,44^ Potential (but unproven) mechanisms modulating the extent of injury to the fetus or newborn infant include viral strain variation; alterations in fetal developmental gene and protein expression; modifications of the placental transcriptome and/or proteome; and post-natal modifications of the infant transcriptome (discussed below; also see Table 3). CMV can be highly neurotropic in the developing fetus, and the virus is readily capable of crossing the fetal blood-brain barrier. CMV replicates in a variety of brain-resident cells including astrocytes, neurons, and microglia.^43,45^ CMV can infect neural precursor cells, in the process inhibiting neuronal differentiation and inducing apoptosis.^46^ Infection of these cells can inhibit normal neuronal proliferation and differentiation of neuronal and astrocytic pathways,^47^ and likely contributes to the cortical malformations of PMG and cerebellar hypoplasia. Injury to the CNS caused by CMV is also highly related to the timing of infection in pregnancy and the corresponding stages of fetal brain development. The most severe brain injuries are associated with first-trimester infections.^5,6^ First-trimester infections disrupt fetal cortical development due to the combined effects of abnormalities in neuronal migration and subsequent cortical organization.^3^ That these events may result in severe abnormalities such as PMG and cerebellar hypoplasia^48^ has been validated in murine^49,50^ and guinea pig^51^ models of CMV infection. Infections later in pregnancy, occurring after the architecture of the fetal brain has been established, may still result in injury including white matter lesions and cysts^4^ and may produce sequelae^52^ even in  the setting of CICMV infections.

**Table 3:** Viral/host factors of potential importance in cCMV pathogenesis/sequelae risk.

**Candidate biomarkers potentially predicting CNS sequelae in cCMV**
**Viral Factors**
• Viral Strain Variation. ◦ Enhanced pathogenicity of specific viral genotypes. ◦ Viral envelope glycoprotein genotypes associated with increased risk of sequelae. ◦ Strain variation/lack of strain-specific immunity promoting immune escape. ◦ Re-infection in seropositives. ◦ Would sequencing infant viral strains/assigning strain genotypes provide prediction of risk of sequelae? • Infection Mediating Immunopathogenesis and Host Immune/Inflammatory Response. ◦ Modification of host cytokine and chemokine responses. ◦ Viral immediate early (IE) gene, anti-apoptotic genes, viral cytokine/chemokine proteins, G-protein coupled receptors, TNFα and interleukin homologs.
**Host Factors**
• Injury to Host Chromosome ◦ 1q23.3/DFNA7 locus. ◦ 1q42/USH2A. • Interaction of CMV with Host SNPs. ◦ Cytokine promoters. ◦ TLR promoters. ◦ Gene-gene interactions, e.g., *GJB2*. • Disruption of Genes that are Key Components of Fetal Brain Transcriptome with Resulting Neuronal Migration Abnormalities. ◦ *LIS1*. ◦ *NID1*. • Systemic Transcriptome Analyses in Children with Sequelae and Unaffected cCMV Controls. ◦ 16-gene “signature” associated with SNHL. ◦ *ARHGEF9*. ◦ *MPDU1*. ◦ *CD40*. ◦ Would measurement of host transcriptional responses provide clues to prognosis and help individual patient management/therapy decision-making? • Host Production of Neurotoxic Cytokines and Reactive Oxygen Species. ◦ Could the measurement of cytokine, chemokine or virus-specific immune responses in infants help to profile risk and to guide prognosis/clinical management?

Beyond the descriptive level, the molecular and cellular pathogenesis of fetal brain injury likely reflects both variations in viral genes and host response to infection. Viral genotypes have been studied as a potential biomarker for increased risk of disease and sequelae. CMV genotypes are typically defined by sequence polymorphisms in envelope protein coding sequences, including the immunodominant glycoproteins B (gB), H (gH), and N (gN). Polymorphisms in gB are of particular interest because the gB protein is the major target of neutralizing antibody response in the context of natural infection and is a leading subunit vaccine target.^53^ Evidence is mixed regarding the comparative virulence of gB genotypes. Of note, some studies in other patient populations with CMV disease suggest that genotype is an important determinant of viral virulence,^54^ while other studies have failed to show such associations.^55^ An increased risk was associated with the gH-1 genotype.^56^ These results suggest that the gH genotype may be associated with SNHL, but not other manifestations of cCMV disease. For gN, Pignatalli et al. found that symptoms at birth, abnormal imaging results, and sequelae were associated with a specific gN genotype, gN-4.^57^ Other viral genes of interest include the *UL144,*^58–61^ and the *UL146/UL147* genes. The search for a definitive CMV genetic marker that portends increased risk for sequelae has been reviewed by Arav-Boger,^62^ who reports that no clear candidate gene has emerged as the key mediator of injury in the context of cCMV infection.

The impact of CMV on host chromosomes was reported in 2000^63,64^ with two specific breaks in chromosome bands 1q42 and 1q21 found. Subsequent fine-mapping of the 1q21 breakpoint localized CMV-induced genetic damage to band 1q23.3 between LMX1A (the DFNA7 locus) and another hearing impairment locus with no gene yet identified (DFNA49). Variants in *LMXA1* have been associated with an autosomal dominant progressive form of SNHL.^65^ The breakpoint was also located close to the MPZ gene previously shown to be involved in autosomal dominant Charcot-Marie-Tooth syndrome, a syndrome associated with auditory neuropathy.^66^ The less studied 1q42 breakpoint is located close to *USH2A,*^67^ a gene associated with Usher syndrome type IIa that is characterized by SNHL and blindness. Although chromosome analysis is not recommended for infants with cCMV infections, these findings warrant additional study. At the individual gene level, CMV is associated with single nucleotide polymorphisms (SNPs) in Toll-like receptors,^68,69^ cytokine gene promoters,^70^ and polymorphisms in gap junction beta 2 (GJB2), more commonly known as connexin 26. This interaction is of potential interest given that autosomal recessive mutations in GJB2 are a common cause of hereditary SNHL.^71^

CMV-mediated alteration in the host inflammatory response is another potential pathway that likely contributes to fetal neuropathogenesis.^72^ CMV infection has been shown to induce cytokines that contribute to neuroinflammation in murine models.^50,73^ Neuroinflammation can be mediated by viral triggers that track through astrocytes, associated with increased production of CCL2, CXCL8, CCL3 and CCL5, as well as reactive oxygen species.^74,75^ Microglia undergo a marked increase in the production of TNF-α and IL-6,^74,76^ as well as chemokines such as IL-10,^77^ which is upregulated by infection of primary microglial cells but not astrocytes. CMV also encodes a plethora of immunomodulatory gene products, including G-protein-coupled receptors, chemokines, cytokine homologs (including IL-10), and a TNF receptor homolog.^78,79^

Beyond the inflammatory response, CMV infection can dramatically modify host transcription,^80^ which also likely impacts viral pathogenesis. The CMV major immediate early gene (IE) locus encodes proteins that have a major impact on host transcriptional responses, and these gene products^81^ as well as the CMV UL36, 37 and 38 transcripts modulate apoptosis.^82^ Of particular interest is the dysregulation of genes and pathways linked to autism spectrum disorder, and other neurodevelopmental disorders in cell culture and organoid models.^83^ CMV-infected neural stem cells demonstrated up-regulation of *PAFAH1B1*,^84^ which encodes LIS1 (lissencephaly-1), a protein important in directing neuronal migration.^85^ CMV down-regulates the nidogen 1 (NID1) protein, which is a basement membrane protein also involved in neuronal migration.^86^ Extending this line of investigation to the patient, Ouellette et al. examined the blood transcriptional profile of 80 infants with cCMV (49 symptomatic, 31 asymptomatic), who were followed longitudinally for the first three years of life. Correlative assessments comparing symptomatic (*n* = 49) and asymptomatic (*n* = 31) infants were undertaken, including assessments for SNHL.^87^ Although overall the biosignatures of symptomatic and asymptomatic cCMV were identical, a 16-gene classifier signature with an accuracy of >90% in correlation with SNHL was identified. Of these genes, *CD40* was of interest, since its expression has been found to be increased in other patient populations with SNHL. Two other genes that contribute to the signature, *ARHGEF9* and *MPDU1*, have been associated with intellectual disability. Two key strengths of the Ouellette study are: (1) the measurement of biomarkers in vivo, and not just in cell culture systems and (2) inclusion of individuals with asymptomatic cCMV. A high-priority area is to extend these analyses to asymptomatic children identified by universal cCMV screening, looking to discover biomarkers that identify the 15–20% of these infants that are at risk for adverse sequelae.

### Neuroimaging

Many infants with cCMV undergo brain imaging as part of standard clinical care. Before reviewing the relevant literature, we first discuss the strengths and weaknesses of cranial ultrasound (CUS) and magnetic resonance imaging (MRI), the two most commonly used brain imaging techniques used to evaluate the neurodevelopment of infants with cCMV. As noted above, CUS is typically the initial screening technique used when cCMV is identified, and was the cornerstone of the evaluation of newborns with asymptomatic cCMV in the pilot screening study in Minnesota that predated initiation of state-wide universal screening.^88^ The technique uses high-frequency sound waves to generate structural images of the brain. Benefits of CUS include it being inexpensive, and low risk. Notably, CUS is widely accessible; it is a portable device that can be used at the patient’s bedside. However, CUS is highly operator dependent, with sonographers using different techniques and settings that can impact the appearance of white matter, making detection of subtle brain abnormalities challenging.^89^ Moreover, since the commencement of universal cCMV screening in Minnesota, we have seen numerous instances in which CUS demonstrates findings that are of uncertain significance in otherwise CICMV cases,^90^ including findings such as cysts and leukostriate vasculopathy (LSV; reviewed in more detail below) that have not been uniformly compatible with the published cCMV disease-defining categorization reported from expert groups.^33–35,88^

MRI uses large and powerful magnets to create highly detailed 3-dimensional images of the body. MRI is considered the gold standard for detecting brain injuries in neonates due to its ability to detect subtle changes, in particular white matter injury, that may be missed with CUS examination.^91^ While MRI generates high quality medical information, collecting the data can be challenging. MRI data is susceptible to motion, the scan environment is loud, and costs are high when compared to CUS. To reduce movement, scans can be collected after the infant is sedated or while they sleep naturally. Sedation is generally considered safe although both short- and long-term potential risks are known.^92^ Collecting MRI data while infants sleep naturally can also be challenging and success rates vary widely across hospitals and research groups.^93–95^ Some approaches to facilitate natural sleep MRI include Half-Fourier Acquisition Single-shot Turbo Spin Echo (HASTE) scans that are shorter in duration, usually less than 1 min, but yield much lower quality data.^96^ Recent advances in MR physics attempt to balance speed with image quality, and include the development of sequences that are both fast (less than 9 minutes for T1w/T2w), high quality (0.8 mm isotropic for T1w/T2w), and less susceptible to motion.^97^

Brain imaging studies—both CUS and MRI—in children with CACMV have shown multiple abnormalities that reflect brain disruption including white matter lesions, calcifications, ventriculomegaly, ventricular adhesions, and in more severely affected children, malformations of cortical development (PMG, heterotopia) and cerebellar hypoplasia.^98–102^ Later studies also describe periventricular pseudocysts (PVPC) and LSV in infants with cCMV.^101^ The nomenclature of these lesions has been inconsistent. PVPC have been given different terms based on their location. Here we will consider frontal horn (connatal), caudothalamic (subependymal pseudocysts) and temporal horn cysts all as examples of PVPC, with caudothalamic the most commonly reported in most studies. LSV is also variably described as necrotizing or thalamostriate vasculopathy in the literature, but we do not attempt to draw any distinction between these classifications.

**Fig. 2:**
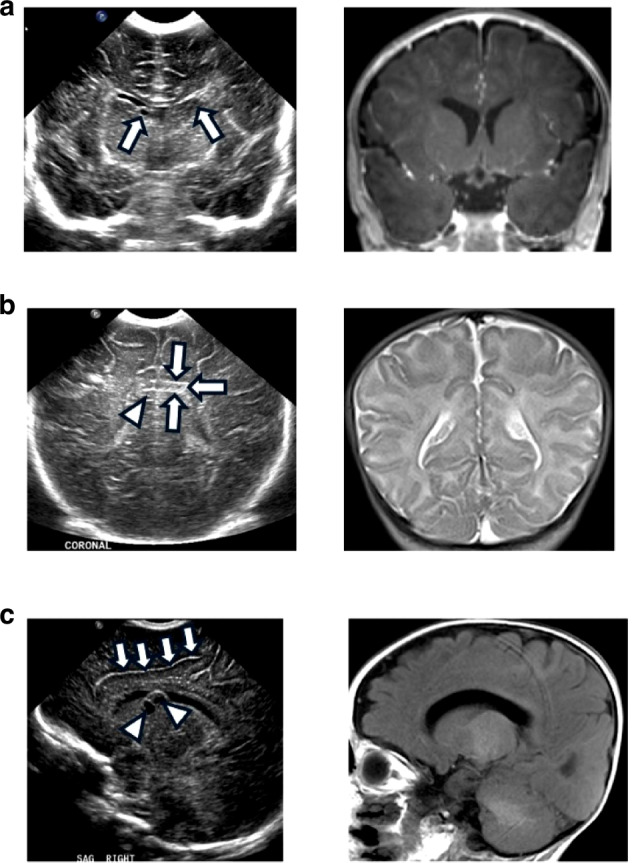
CUS findings of LSV and/or PVPCs do not always predict MRI abnormalities in CICMV. This image demonstrates brain MRI abnormalities seen in CICMV infections in children identified by universal cCMV screening. **a** Images from a child with CICMV with findings of bilateral subependymal cysts (left panel, arrows) by coronal image CUS, but normal MRI findings (right panel) on a follow-up MRI evaluation performed 4 months following the CUS. **b** Images from a child with CICMV with findings of left-sided subependymal cyst (arrowhead) and suspected scattered punctate echogenic foci (arrows); these were interpreted as possibly representing calcifications in the setting of CMV versus artifact. Follow-up contemporaneously ordered MRI demonstrated normal MRI findings (right panel). **c** Left panel, bilateral subependymal cysts noted (arrowheads) on sagittal view in infant with CICMV, with LSV (arrows) within both basal ganglia. Brain parenchyma was interpreted as otherwise normal in echogenicity and echotexture. Right panel, brain MRI obtained 6 months following CUS was interpreted as normal.

Brain imaging studies in children with CICMV are fewer but show the same abnormalities reflecting brain disruption as seen in individuals with CACMV although at lower frequencies. For example, white matter lesions and ventriculomegaly were found in 76.9% and 46.2% of newborns with CACMV compared to 30.9% and 4.2% in those with CICMV.^101^ Similarly, PVPCs were found in 61.5% of newborns with CACMV compared to only 15.8% in those with CICMV with both rates higher than found in healthy cohorts. The significance of PVPC has remained unsettled, although we note that these lesions were seen in only 4.8% of a large cohort of mostly healthy neonates.^103^ In the same study, detection of multiple PVPCs was associated with an increased risk of an abnormal outcome. These imaging studies lack neurodevelopmental follow-up, but the broader literature on neurodevelopmental sequelae of PVPCs suggests that CICMV may be a risk factor for abnormal neurodevelopmental outcomes. In a pilot study in Minnesota from 2016 to 2022, PVPCs were commonly encountered in infants with CICMV.^88^ They have also been commonly noted in newborns with CICMV since the advent of universal cCMV screening in 2023.^90^ The key concern in this context is that a normal CUS examination can provide a false sense of reassurance in the evaluation of a CICMV infection, insofar as a MRI may be abnormal when the screening CUS is read as normal.^88^ Alternatively, PVPCs and LSV may still represent incidental findings in CICMV infections in children who are not – given our current level of knowledge—clearly at risk for sequelae, nor candidates for antiviral therapy. Indeed, we have observed that some infants with PVPCs or LSV incidentally identified by CUS in infants identified by universal screening have had normal brain MRIs (Fig. 2). We provide recommendations below regarding which CICMV infants identified by universal screens should undergo MRI.

A number of reports have directly examined the relationship between neonatal brain imaging studies and neurodevelopmental outcome in infants and children with congenital infection. Neonatal brain imaging and neurodevelopmental status with follow-up for 12 months or longer were reported for 26 infants with symptomatic cCMV (CACMV).^104^ The population consisted of all neonates with symptomatic cCMV consecutively admitted to La Paz Hospital, a University Tertiary Hospital located in Madrid, Spain, from 1993 to 2009. In this study, the authors described a 4-level neuroimaging score (Table 4) determined by radiological review of CNS imaging studies which included CUS (25/26 infants), CT (11/26) and T1- and T2-weighted MRI scans (9/26). This scale was based on criteria from Noyola et al., who previously described a validated neuroimaging scale comprising calcifications, ventriculomegaly and atrophy,^105^ with modifications. Specifically, this scale was modified by adding cerebral dysgenesis and white matter disease, with findings graded on a scale of 0–3.^104^ The investigators found that both scales showed a significant association with outcome, with the new scale demonstrating increased accuracy in predicting death or moderate-to-severe disability. The novel scale was highly associated with outcome prediction by MRI, but was less useful in predicting an unfavorable outcome in 2 patients with mildly abnormal CUS findings. The authors concluded that while a strictly normal interpretation of CUS predicted a favorable outcome, in case of subtle US abnormalities, MRI was more useful for establishing prognosis.

**Table 4:** Scoring system as defined by Alarcón et al. for assessing cCMV neuroimaging abnormalities.^104^

Score	Description
0	None of the following abnormalities.
1	Single punctate periventricular calcification, lenticulostriate vasculopathy, caudothalamic germinolysis, ventriculomegaly (excluding severe)^a^ and focal/multifocal white matter signal abnormalities on MRI.
2	Multiple discrete periventricular calcifications, paraventricular germinolytic cysts, severe ventriculomegaly, diffuse white matter signal abnormalities and/or temporal lobe involvement.
3	Extensive calcifications, brain atrophy, abnormal gyration, cortical malformation, dysgenesis of the corpus callosum and/or cerebellar hypoplasia.

^a^Ventriculomegaly was defined as ventricular index exceeding the 97^th^ centile for gestational age and considered severe when the ventricular index surpassed the 97^th^ centile plus 4 mm.

The relationship between neonatal brain imaging studies and neurodevelopmental outcome was further explored in a retrospective cohort study of 160 infants with cCMV reported from 8 European university hospitals.^106^ In this study, 103 infants were defined as symptomatic, 55 were asymptomatic, and 2 were of indeterminate status, using the Rawlinson et al. criteria.^33^ Information about neonatal brain imaging and neurodevelopmental status (with follow-up for 12 months or longer) was reported for 45 infants in the asymptomatic cCMV (CICMV) group. The basis for neonatal diagnosis of cCMV in this sample was variable, and included infants identified because of maternal seroconversion during pregnancy. In this study, 85% of infants with asymptomatic cCMV (as described by the authors) had at least one neuroimaging abnormality. The most common were white matter abnormalities, observed in 45% of the asymptomatic cCMV MRIs. According to the authors’ classification system, only 13% were scored as having no abnormalities (score of 0); 60% had a single destructive lesion, teratogenic abnormality, or white matter abnormality (score of 1); 27% had multiple lesions or abnormalities (designated by a score of 2); and none were scored a 3 (which would have indicated extensive abnormalities). A normal developmental outcome was found in 87% of the asymptomatic cCMV infants. Of the 13% infants who had disability or severe outcomes, all also had neuroimaging abnormalities identified upon radiological review.

As noted above, this same study included infants with symptomatic cCMV as well and found that moderate or severe neuroimaging abnormalities, in particular those with white matter findings, were associated with a high risk of adverse sequelae.^106^ Notably, infants with higher qualitative neuroimaging scores, especially those with white matter abnormalities seen on review, also had higher apparent diffusion coefficient (ADC) values in frontal, parietal, and temporal regions when compared to those without white matter abnormalities. ADC values are a measurements of water diffusion; during early brain development ADC values decrease as cell membranes, myelinated axons, and extracellular molecules develop and become more structurally coherent. Therefore, higher ADC values in infants with cCMV with white matter abnormalities may reflect delayed or atypical brain development. Notably, higher ADC values were found in all 6 brain regions tested even though white matter abnormalities were typically more restricted.

Finally, the most recent report we reviewed analyzed neonatal brain MRI with diffusion-weighted imaging in 255 individuals with cCMV (combining CACMV and CICMV) and obtained quantitative measurement of ADC in several regions of interest placed across the brain. The primary result showed that white matter ADC in regions of interest was significantly higher in children with cCMV who also had comorbid neonatal hearing loss, cognitive impairment, or motor impairment when compared to cCMV infants without these conditions.^99^ ADC values were used to predict neonatal hearing loss, cognitive impairment, and motor impairment in the sample of cCMV infants, with results demonstrating some clinical utility with respect to the ability of white matter findings to predict outcomes. We recommend that future research should build on this work by quantifying white matter development across the entire brain (as opposed to limited regions of interest) and by leveraging recent advances in diffusion MRI in quantifying white matter fiber bundles.^107^ However, brain imaging studies in cCMV conducted to date all have significant limitations. Most have been retrospective qualitative analyses of clinically acquired MRI or CUS. While such studies are an excellent use of existing data, they are limited. Variations in scanner model, magnet strength and imaging sequences all make harmonizing data difficult, which in turn limits quantitative approaches. Of critical importance is that many studies lack a healthy (uninfected) control group from which to compare results.^107^

Despite these limitations, the collective results are compelling and indicate atypical brain development does occur in many CICMV infants. If confirmed, these results suggests that neonatal brain MRI with diffusion-weighted imaging will likely prove better than CUS or standard T1- or T2-weighted MRI. For now, we recommend a brain MRI study in most newborns with a confirmed cCMV infection.^101^ Table 5 summarizes our recommendations for the specific situations in which brain MRI is warranted in the setting of cCMV, including CICMV. Whether brain MRI should be performed in all cases of cCMV (including CICMV infection with no evidence of CMV disease after diagnostic evaluation) requires further cost-benefit analysis. The brain MRI should be done using standard sequences, not the rapid HASTE sequences used in many clinical circumstances.

**Table 5:** Recommendations for infant MRI scan in setting of cCMV infection.

**Recommendations for MRI Scan in Infants with cCMV**
**Recommended**
• Maternal history of primary CMV infection (seroconversion/virologically confirmed maternal CMV disease) or documentation of maternal re-infection in pregnancy. • cCMV in the context of prenatal ultrasonography abnormalities. • History of maternal valacyclovir therapy for prevention of cCMV transmission. • Small for gestational age or microcephaly. • Asymptomatic cCMV disease (as defined by Table 1) with isolated SNHL. • Ophthalmologic abnormalities. • Any screening cranial ultrasonographic abnormality. • Moderate-to-severe symptomatic cCMV (as defined in Table 1).
**Consider**
• Progressive emergence of SNHL on serial audiological evaluation in infancy. • Abnormal head growth. • Abnormal neurodevelopmental milestones. • Any case with mildly symptomatic cCMV disease (as defined in Table 1). • Infants with asymptomatic cCMV infection (as defined in Table 1) on a case-by-case basis.

### Neurodevelopmental assessment

As noted above, children with CACMV are at risk for a wide range of neurodevelopmental sequelae, including intellectual disability, cerebral palsy, seizure disorders, learning disabilities, cerebellar dysfunction, and vision impairment from retinal disease, cortical blindness or both. A recent systematic review reported global developmental delay in 46–64% of children with CACMV.^108^ The situation is less clear for infants with CICMV. Inconsistencies in the definitions of “symptomatic” and “asymptomatic” congenital infection, differences in the types of developmental and neurocognitive assessments used, and, in some studies, the absence of control groups or norm referenced tests, have all complicated our understanding of long-term sequelae in CICMV.^109,110^ Notably, the definitions of cCMV (e.g., symptomatic vs. asymptomatic) historically have not included cognitive or developmental status.

In general, the literature on developmental outcomes in CICMV is reassuring. A recent study of 253 neonates with cCMV found that if children had normal hearing at birth, normal platelet count, and a normal cranial ultrasound, the risk of neurologic sequelae was not increased.^111^ When examining studies that have utilized standard, norm-referenced developmental or cognitive measures with a control group, no early developmental group differences between CICMV participants and controls have been found. A recent systematic review of neurodevelopmental outcomes in children with “asymptomatic” cCMV from studies published between 2016-2022 identified nine studies that compared outcomes of CICMV and uninfected children.^110^ Two studies showed no differences in cognitive/developmental skills on the Bayley Scales of Infant and Toddler Development between 6 and 24 months.^112,113^ A third study used the Griffiths Mental Development Scales and similarly found no differences between asymptomatic CMV and controls at 18 months,^114^ while a fourth found no differences in parent-reported milestones and diagnoses within the first two years.^115^ Of note, most of these studies excluded children with SNHL from their “asymptomatic” group. More recently, Stoyell et al. found no group differences on a standard developmental measure (Mullen Scales of Early Learning), or parent-reported questionnaires rating early social and emotional development (ITSEA), between 29 asymptomatic cCMV participants (identified through universal screening) and sex and age-matched controls at 12 months of age.^116^

Five other studies, with overlapping cohorts, followed children for more than five years. The first set followed 107 children with asymptomatic cCMV and 274 matched uninfected children through age six and found no differences in cognitive or speech language impairments documented in their medical records or from parent reports on the Child Development Inventory across domains (e.g., cognitive, motor, language, social).^29,117^ However, they did find that a higher proportion of children with asymptomatic cCMV had motor impairments documented in their medical chart. The Houston Congenital CMV Longitudinal Study followed asymptomatic cCMV participants across middle childhood and adolescence, and found no differences on global delays (unspecified measures), parent-reported attention or impulsivity (using the Behavior Assessment System for Children or “BASC” exam), expressive vocabulary, or academic achievement.^118–120^ Specifically regarding SNHL, Lopez et al. examined IQ scores across later childhood and adolescence (up to age 18) and found no differences between children with asymptomatic cCMV without SNHL (by 2 years) and controls, but found lower scores in a subset of children with asymptomatic cCMV and SNHL on measures of full scale IQ and receptive vocabulary, but not on measures of verbal and nonverbal intelligence, expressive vocabulary, and academic achievement in math or reading.^119^

Despite these largely reassuring results, concerns persist that children with CICMV may struggle with executive functioning skills, although this has not always born out in research.^121,122^ A recent cross-sectional study of 24 children with asymptomatic cCMV (average age 5.5 years) used the Child Behavior Checklist (CBCL) assessment to measure parent-reported emotional and behavioral challenges.^123^ The CBCL found that 5/24 children (20.8%) with asymptomatic cCMV had clinically elevated scores for affective, ADHD or oppositional defiant problem subscales, while behavioral/emotional problems were noted in 15/41 (36.6%) of children with symptomatic cCMV. In contrast, in a study of older school-aged children with asymptomatic cCMV infection (*n* = 76) using the BASC these children were not at increased risk for hyperactivity or attention problems.^120^ In a smaller study that examined attention and executive functioning in a sample of children with cCMV (with no other neurological diagnoses) and SNHL treated with cochlear implant surgery (*n* = 10), the children (ages 4–13 years) were found to have lower phonological working memory scores, but were rated similar to controls on parent-reported executive functioning skills, using the Behavior Rating Inventory of Executive Function (BRIEF) assessment tool. The sample was too small to examine differences between the groups by age in other direct measures of attention or executive function.^121^

One particularly important and emerging area of study is the question of whether cCMV could be associated with autism spectrum disorder. Possible links between autism and CMV have a 40-year history. Autism is a lifelong neurodevelopmental condition that is clinically characterized by differences in social communication and the presence of restrictive or repetitive behaviors.^124^ Early social, behavioral, and cognitive markers of autism are evident within the first two years of life^125,126^ and can present as early as 9 months of age in infants later diagnosed with autism.^127^ Current estimates suggest that the prevalence of autism is 1 in 36 children in the US.^128^ The causes of autism are unclear with evidence for both genetic and environmental contributors. Genetic contributions to autism risk are highly variable, with over 100 genes meeting rigorous thresholds, almost all associated with more severe forms of autism. This number continues to grow as larger studies are conducted.^129,130^ Common variants are likely to account for most of the genetic risk, especially for less severe forms, although a few rare variants have been discovered.

Few studies have been reported that have focused on viral infections as a possible contributor to autism. Theoretically, it is possible that viral infection during pregnancy could contribute to perinatal inflammation, which in turn could impact gene expression. It is also possible that cCMV infection could cause direct damage to the developing CNS resulting in autism-like symptoms.^131^ The majority of studies investigating the links between cCMV and autism are single clinical reports (see Supplementary Table 3 in Pesch et al.^132^ for a comprehensive review). However, a few noteworthy studies include data from larger samples and use approaches of mining medical records for information on cCMV and autism,^132^ testing neonatal dried blood spots for cCMV and then mining records for autism information,^29^ and prospectively following infants from a cCMV registry and using records for autism diagnostic information.^133^ Pesch reviewed Medicaid claims from the years 2014–2020 for nearly 3 million children and evaluated rates of cCMV and autism, with results indicating that children with cCMV were more than 2.5 times more likely to have autism (hazard ratio: 2.5) when compared to children without cCMV.^132^ Due to the nature of the available data, the researchers were unable to classify participants as having symptomatic vs asymptomatic cCMV, although they speculated that most of the children likely had symptomatic cCMV. In the recently reported study by Keymeulen et al.^133^ that makes use of the Flemish cCMV registry^134^ neurodevelopmental outcomes in infants with cCMV were investigated. Data was available for 753 infants, many of whom were identified as having cCMV after their mothers were screened for seroconversion at 12–13 weeks of gestation. The children were followed longitudinally by centers providing clinical care and results indicated that 2.6% of the cCMV children were diagnosed with autism, which was higher than the prevalence of autism in Flanders at the time (0.6–0.7%). The last study to be highlighted took a different approach and tested 31,484 neonatal dried blood spots for cCMV. Medical records were reviewed and results indicated that 3% of infants with cCMV also had autism. Interestingly, the rates of autism were higher in symptomatic cCMV when compared to asymptomatic cCMV (7.7% versus 1.9%, respectively).^135^ The results from these highlighted studies are in line with those from a 2017 meta-analysis which found an increased prevalence of autism in cCMV (odds ratio of 11.31).^131^

The mechanism behind a potential increased risk for autism in infants with cCMV is unclear, but at this point we cannot exclude the possibility that the increased developmental surveillance of infants with cCMV may partly contribute to increased prevalence. It does seem clear based on existing data that cCMV does not result in autism in the vast majority of affected individuals. Likewise, most children with autism did not have cCMV as infants. Prospective studies that include universal screening and long-term follow-up, including assessments of executive function and cognitive performance through early childhood, are needed to better understand the relative risk of autism for infants with cCMV. If the proposed links between cCMV and autism can be confirmed, it is conceivable that a successful maternal vaccine program could reduce the frequency of autism in childhood.^136^

In summary, when we examine studies that utilized control groups and/or norm-referenced measures, the broad picture of neurodevelopmental outcomes in asymptomatic cCMV has been largely reassuring. However, limited longitudinal data, small sample sizes, overreliance on broad-stroke measures/outcomes, inconsistent definitions, and a paucity of studies utilizing uninfected controls all continue to impair our understanding of developmental outcomes in this population.

## Conclusion

### Summary, recommendations, and key areas for future research

Congenital CMV is the most common perinatally-acquired infectious disease responsible for childhood disabilities. Infants with cCMV are at risk for neurologic and developmental disabilities. The likelihood of long-term disability is higher with evidence of CMV disease at birth, which occurs in around 20% of newborns. However, some children with cCMV do not present with evidence of the disease at birth, but nonetheless will manifest with long-term sequelae later in childhood (generally SNHL). The goal of universal screening for cCMV is in part to identify this group of children. Minnesota is the first US state where the state Department of Health has implemented universal screening for cCMV, but other states are following. New York and Connecticut are pursuing universal cCMV screening^39^, and two Canadian provinces (Ontario and Saskatchewan) have commenced universal screening programs.^137^ Data from universal screening in Minnesota unexpectedly found many infants classified as being “asymptomatic” for cCMV at birth had non-classical neuroimaging findings, including white matter lesions and subependymal pseudocysts of unclear significance.^88^ Studies by others attribute a variety of non-classical CNS sequelae to cCMV, including postural asymmetry and atypical body movements,^138^ autism spectrum disorders,^132^ and challenges related to attention and behavior regulation.^123^ However, other studies have reported no increased risk for impairment of cognition skills.^119,139–141^ Given the potential relationship between neuroimaging findings and neurodevelopmental outcome, future studies should examine these issues in tandem. In this context, animal models of cCMV need to be studied in parallel with human clinical studies. Studies in rhesus macaques,^142^ guinea pigs,^143,144^ and mouse models,^145^ each using the respective species-specific CMV, can recapitulate much of the pathology observed in infants, and provide useful systems to explore pathogenesis and vaccines designed to prevent cCMV infection.

Resolving the discrepancies in the literature is of utmost importance as clinicians continue to face the challenging clinical management problem of coordinating care recommendations and crafting developmental surveillance plans for infants who screen positive for cCMV at birth. As more states in the US and provinces in Canada incorporate universal cCMV screening into newborn screening programs, the management uncertainties will compound.

To address key knowledge gaps impacting clinical care for infants with cCMV, the field requires a well-controlled, highly-powered, and unbiased prospective study of newborns who screen positive for cCMV at birth. It is paramount that this study be conducted in an environment where universal screening is in place to avoid ascertainment bias. The study should also include a contemporaneous control group of infants who were also screened but results were negative. Such a study would provide much needed information on the rates of developmental delay, neurological abnormalities, SNHL, and behavioral challenges for infants who have cCMV at birth but are classified as asymptomatic. A clear picture of developmental risk patterns is essential to crafting clinical recommendations for treatment and neurodevelopmental surveillance plans. Delineation of the heretofore largely unknown long-term risks of CICMV infections, beyond hearing loss, would have substantial public health implications in making decisions about the advisability of adoption of universal cCMV screening as a public policy recommendation.
